# Finite Element Modeling for Debonding of FRP-to-Concrete Interfaces Subjected to Mixed-Mode Loading

**DOI:** 10.3390/polym9090438

**Published:** 2017-09-09

**Authors:** Hui Yu, Yu-Lei Bai, Jian-Guo Dai, Wan-Yang Gao

**Affiliations:** 1 Key Laboratory of Urban Security and Disaster Engineering of Ministry of Education, Beijing University of Technology, Beijing 100022, China; yuhuibjut@163.com; 2 Department of Civil and Structural Engineering, The Hong Kong Polytechnic University, Hong Kong 999077, China; cejgdai@polyu.edu.hk; 3 School of Naval Architecture, Ocean and Civil Engineering, Shanghai Jiao Tong University, Shanghai 200240, China; gaowanyang20022003@gmail.com

**Keywords:** FRP-to-concrete interface, mixed-mode loading, finite element analysis, cohesive zone model, cohesive element, peeling angle

## Abstract

This paper presents finite element (FE) modeling of the debonding behavior of fiber reinforced polymer (FRP)-to-concrete interfaces subject to mixed-mode loading, which is realized through a peeling test of FRP composites externally bonded onto a concrete substrate. A cohesive zone model (CZM) is implemented into the FE model to represent the behavior of the FRP-to-concrete interface. Two element schemes (orthotropic plane stress element and beam element) were employed to simulate the behavior of FRP composite plate in the peeling test. The orthotropic plane stress element scheme, bearing a clear physical background and with an easy definition of the material property parameters following the composite mechanics, is found to be superior to the beam element scheme, and thus is utilized to conduct parametric studies. The influences of the peeling angle, the interfacial parameters (i.e., the configuration of the cohesive zone models, the interfacial damage initiation law (DIL), the interfacial damage evolution law (DEL), the coupling of mode-I and mode-II components), on the mixed-mode failure of the FRP-concrete-interface are carefully investigated. The results showed that the mode I component plays a critical role in the debonding failure of FRP-to-concrete interfaces even when the peeling angle is very small. The failure of FRP-to-concrete interface transits promptly from a mode II-dominated one to a mode I-dominated one when the peeling angle increases to a relatively small value (e.g., 4 degree) and subsequently the peeling force (i.e., the debonding strength of FRP) decreases dramatically. Such mixity of the mode I and mode II components should be appropriately considered for refining the analysis of FRP-strengthened RC beams and the FRP debonding strength design, for which a pure mode II interfacial failure was usually assumed.

## 1. Introduction

Externally bonded fiber reinforced polymer (FRP) plates or sheets are now widely recognized as an effective technique for strengthening reinforced concrete (RC) structures [1,2,3,4]. In concrete members strengthened with externally bonded FRP composites, debonding of FRP-to-concrete interfaces usually is a dominant failure mode particularly for those bond-critical applications (i.e., flexural and shear strengthening). A good understanding has been achieved on the various debonding failure modes in FRP-strengthened RC beams (e.g., [1,2]). These failure modes were usually classified according to the failure mechanisms/appearances of FRP-strengthened RC members. According to the stress conditions to which the FRP-concrete interface is exposed, the debonding failure of the FRP-to-concrete interface in fact can be simply classified into three categories: mode I, mode II and mixed-mode failure (e.g., [5]), which correspond to an interface subjected to a tension action, a shear action and a combined action of tension and shear, respectively. Since adhesives used to bonding FRP composites are usually much stronger than concrete, the above three types of interfacial failure are in fact caused by the tensile failure of concrete, the shear failure of concrete and a combination of both from a macroscopic view.

Debonding of FRP in FRP-strengthened RC members is associated either with the discontinuity of FRP composites (i.e., the FRP cut-off location) or with the discontinuity of concrete (i.e., the crack locations). Both lead to normal and/or shear stress concentration at the FRP-to-concrete interface. This paper deals with the latter discontinuity, which is believed to be more critical, since it is impossible to utilize efficiently the FRP strength while keeping the concrete un-cracked unless the concrete is prestressed.

**Figure 1:**
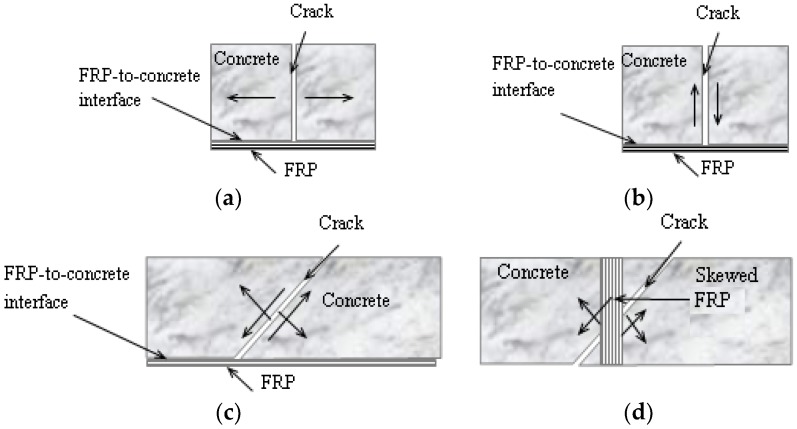
Debonding of fiber reinforced polymer (FRP)-to-concrete interfaces in bond critical applications: (**a**) mode II failure; (**b**) mode I failure; (**c**) Mixed-mode failure; (**d**) Debonding of skewed FRP.

In a plane FRP-strengthened RC member, a concrete crack may exhibit two different types of displacements: one is the opening of the crack (Figure 1a), which is vertical to the crack direction and parallel to the FRP plane. This may occur at a constant moment zone of the strengthened member and causes shear slips at the FRP-to-concrete interface, leading to a global mode II interfacial failure; the other is the shear sliding of the crack (Figure 1b), which is parallel to the crack direction and vertical to the FRP plane. This may occur in the case that FRP composites are bonded to the concrete soffit to prevent its spalling (e.g., [6,7,8]) and causes opening displacements at the FRP-to-concrete interface, leading to a mode I interfacial failure. A more common case is that a crack exhibits the above two types of displacements simultaneously (i.e., the member is subject to a combined action of flexure and shear). In this case, the FRP-to-concrete interface is subject to a mixed-mode failure (Figure 1c). One thing to note is that the fiber orientation in FRP composites is usually vertical to the crack direction in flexural strengthening cases, so that the tensile stiffness and strength of fibers can be utilized most efficiently. However, this may not be true when the FRP composites are used for shear strengthening, in which case the fiber orientation may not coincide with the crack opening direction (Figure 1d). As a result, the opening and the sliding of concrete cracks produce interfacial slips parallel to the FRP plane both along and perpendicular to the fiber orientation. Dai and Cao [9] defined such an FRP as a skewed FRP and studied the skew effect on the debonding strength of FRP composites. However, since concrete cracks do not cause the opening displacement perpendicular to the FRP plane, the debonding failure of the skewed FRP can be regarded as a biaxial shear (mode II)-induced failure at the FRP-to-concrete interface rather than a mixed-mode one, and hence is not discussed in this study.

Among the three types of debonding failure, the mode II failure of the FRP-to-concrete interface has been studied most extensively (e.g., [10,11,12,13,14,15,16,17,18,19,20,21,22]) through experimental and analytical studies. Although the mode I failure was usually ignored for the design of FRP strength for crack-induced debonding in the past, its fracture behavior has also been explored by limited research (e.g., [23,24,25,26]) through three-point bending tests on notched concrete beams incorporating an FRP-to-concrete interface.

In recent years, there have been increasing experimental tests (e.g., [6,7,8,16,27,28,29,30,31,32]) and numerical analyses on the mixed-mode failure of the FRP-concrete interface based on either a closed-form solution (e.g., [13,33,34,35,36,37,38,39,40]) or a finite element (FE) analysis (e.g., [31,41,42,43,44,45,46,47,48]). Two major issues remain controversial on the bond modeling of the FRP-to-concrete interface subject to mixed-mode loading. The first issue is the quantitative relationship between the peeling angle and the interfacial bond strength, in particular, when the peeling angle is small, which is the case for FRP flexurally-strengthened RC beams. It is generally believed that the shear deformation of the strengthened beam is much smaller than its flexural deformation at the ultimate states. As a result, the effect of mode I component is usually ignored in the current code of practice when predicting the mode II debonding strength of FRP in FRP-flexurally strengthened RC beams. However, this may not be true because even a very small shear deformation may lead to a large peeling angle locally and consequently a mode I dominant FRP debonding failure at the toe of any diagonal crack (i.e., a flexural/shear crack), since the ability of the FRP-to-concrete interface in tolerating the mode I deformation is weak. Eventually the debonding failure may become mode II dominant once the peeling angle approaches to zero or an unknown small enough value. This full-range (i.e., at different peeling angles) debonding behavior needs to be carefully examined. In addition, the above transition process is influenced by the FRP property as well (e.g., FRP sheets or laminates with different bending stiffness), which was never explored. The second issue is how the normal and tangential traction-separation laws of the FRP-to-concrete interface should be configured for a mixed-mode loading condition. Although it is difficult to calibrate such laws directly from experimental tests, it is possible to make clear how the initiation and the evolution of the FRP debonding are influenced by the mode I and mode II traction-separation laws and how significant their coupling effects are through numerical analyses. The objective of the current paper is to tackle the above-mentioned two issues through a careful finite element (FE) analysis on a typical peeling test for the FRP-to-concrete interface.

## 2. Peeling Test and Linear Elastic Fracture Mechanics (LEFM) Analysis

The peeling test is a conventional test method in which a thin film is bonded to a substrate and pulled from it at a certain angle (referred to as “peel angle”). As a result, the interface is subject to both normal and shear stresses, leading to a mixed-mode fracture condition. This test has been popularly used to evaluate the fracture properties of a bondline (i.e., in an adhesive or a bond interface between the adhesive and the substrate) in mechanical and material engineering (e.g., [49,50,51,52,53,54]). Lots of experiments have shown that the mixed-mode fracture propagation of the bondline is governed by the critical energy release rate, which is the characteristic of the bondline and independent of the peeling angle unless it is very small [55]. Usually, a large peeling angle corresponds to a pure mode I fracture and a small peeling angle is involved with a high degree of mode mixity.

**Figure 2:**
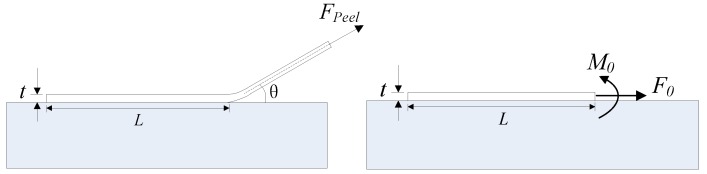
Schematic of a peeling test.

Based upon the LEFM assumption, the mode I and mode II energy release rates, *G_I_* and *G_II_*, during the mixed-mode debonding of FRP-to-concrete interfaces can be calculated as follows [54]:

(1) $$G_{I}=\frac{6M_{0}^{2}}{Et^{3}}\;G_{II}=\frac{F_{0}^{2}}{2Et}$$

(2) $$\;M_{0}=\sqrt{\left[\frac{Et^{3}}{6}\cdot \frac{F_{peel}{}^{2}\sin^{2}\theta }{2Et}+F_{peel}(1-\cos\theta )\right]}\begin{array}{cc} & F_{0}=F_{peel}\cos\theta \end{array}$$

where *F*_0_, *M*_0_ are the mode II force component and the moment in the FRP at the tip of the interface crack; *F_peel_* is the peeling force (Figure 2); *E* is the elastic modulus of FRP; *t* is the thickness of FRP; and *θ* is the peel angle between the FRP and the concrete substrate.

A fracture energy-based failure envelope is needed to govern the mixed-mode fracture propagation of FRP-to-concrete interfaces. Two typical failure criteria have been adopted by previous researchers for FRP-to-concrete interfaces subjected to mixed-mode loading [37,45]:

(3) $$G_{I}/G_{1f}+G_{II}/G_{2f}=1$$

(4) $${\left(\frac{G_{I}}{G_{1f}}\right)}^{2}+{\left(\frac{G_{II}}{G_{2f}}\right)}^{2}=1$$

where *G_1f_* and *G_2f_* are the interfacial fracture energies under pure mode I and mode II conditions, respectively. Obviously, with the combined use of Equations (1)–(3) (or Equation (4)), the relationship between the peeling force *F_peel_* and the peeling angle *θ* can be obtained. However, the closed-form solutions presented above can only be used to predict the steady state peeling force of the FRP-to-concrete interface, as explained later. In addition, the nonlinearity of FRP-to-concrete interfaces, which has been proven to be very significant particularly when the mode II loading effect is predominant, cannot be appropriately considered in the above solutions.

## 3. Finite Element Modeling of the Peeling Test 

**Figure 3:**
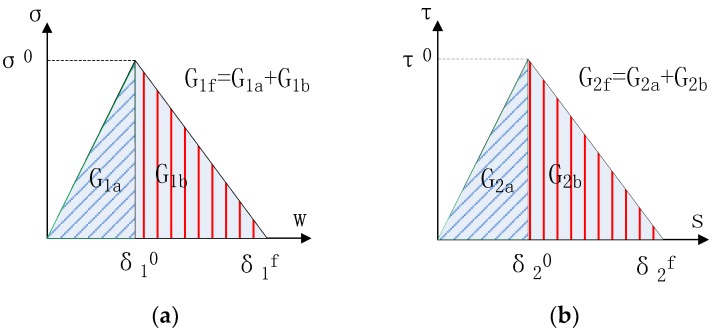
Cohesive zone models for mode I and mode II interface debonding: (**a**) traction-separation law in normal direction (mode I); (**b**) traction-separation law in tangential direction (mode II).

A two-dimensional (2D) FE model is employed in the current paper to simulate the peeling test to overcome the limitation of the closed-form solutions. The FRP elements are directly connected to the fixed boundaries through cohesive elements. The interface is dimensionless and its relative displacement represents the overall damage of the bonding layer including that of the thin layer of concrete adjacent to the bonding adhesive. Therefore, the concrete substrate itself is assumed to be rigid where no damage occurs. When the stiffness of concrete substrate is much larger than that of FRP lamina, this assumption is rational and this assumption has been widely used in previous studies [14,17,37]. A most often used bilinear model is used to describe the cohesive behavior of the FRP-to-concrete interface at both normal and tangential directions (Figure 3). Two different criteria are used to evaluate the debonding initiation of the FRP-to-concrete interface. One is to assume that the mode I and mode II debonding initiations are independent with each other [33]. In other words, the peak local normal stress or the peak local shear stress achieved in the interface under a mixed-mode loading is the same as the value achieved under a single mode I or mode II loading. As a result, the debonding initiation law of the FRP-to-concrete interface can be expressed as:

(5) $$\max\left\{\frac{<\sigma >}{\sigma^{0}},\frac{\tau }{\tau^{0}}\right\}=1$$

where *σ* and *τ* are the peak normal stress and shear stress in the interface at the initiation of the mixed-mode debonding; *σ*^0^ and *τ*^0^ are the peak normal stress and shear stress in the interface at the debonding initiation of the interface under a single mode I and mode II loading, respectively. The other criterion is to assume that the normal and shear stresses have coupled effects and the mixed-mode interface debonding will initiate when the following stress condition is reached: these two equations are referred to as damage initiation law (DIL) hereafter.

(6) $${\left(\frac{\sigma }{\sigma^{0}}\right)}^{2}+{\left(\frac{\tau }{\tau^{0}}\right)}^{2}=1$$

Similarly, the two damage evolution laws (DELs) (i.e., Equations (3) and (4)) introduced above are used in the FE analysis for comparative studies.

**Figure 4:**
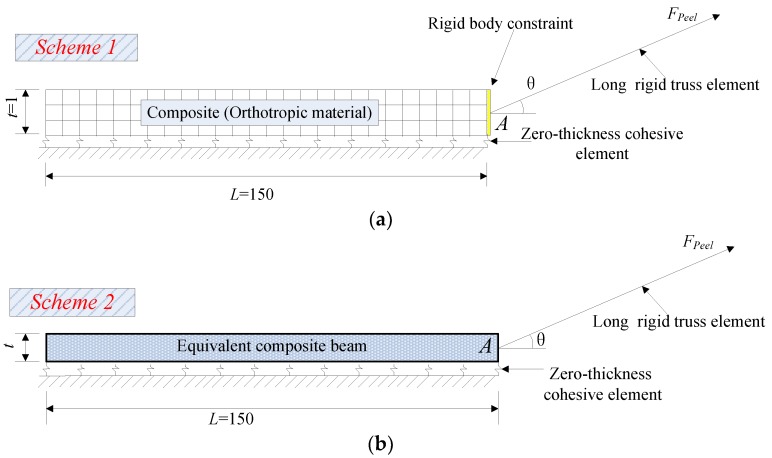
Illustration of the finite element (FE) model of the peeling test (unit: mm): (**a**) Orthotropic 4-node plane stress element; (**b**) 2-node linear elastic beam element.

In the FE model, two element schemes are adopted to simulate the FRP lamina, i.e., orthotropic 4-node plane stress element, and 2-node linear elastic beam element, as used for the above LEFM analysis (Figure 4). In order to make sure that the peel angle remains constant during the whole loading process, the end of the FRP lamina is connected to a truss element with very large axial stiffness and length [33]. Then, the displacement is applied to the far end of the truss element rather than directly to the end of FRP lamina (Figure 4). A rigid body constraint is used in Scheme 1 to make sure all the nodes in the leftmost row can rotate together to comply with the plane section assumption (Figure 4a). The discretization of the geometry is appropriately refined to yield mesh-independent results. All the models (i.e., Equations (3)–(6)) are coded into the finite element analysis platform ABAQUS [56] through defining the user subroutine [48] and the common Newton-Raphson method is adopted to solve the problem.

A unidirectional FRP lamina can usually be treated as an orthotropic material whose mechanical properties in the fiber direction are different from those in the other two orthogonal directions. That is, the elastic modulus, shear modulus and Poisson’s ratios are different in different directions. In Scheme 1, the FRP lamina is modeled as plane stress element, and the mechanical properties of the FRP lamina (i.e., the longitudinal Young’s modulus *E*_1_, the transverse Young’s modulus *E*_2_, the major Poisson’s ratio *v*_12_ and the in-plane shear modulus *G*_12_) can be obtained from the two constituents (i.e., fibers and epoxy) and their volume fractions based on the mechanics of materials approach [57] as follows:

(7) $$E_{1}=E_{f}V_{f}+E_{m}V_{m}$$

(8) $$\nu_{12}=V_{f}\nu_{f}+V_{m}\nu_{m}$$

(9) $$E_{2}=\frac{E_{f}E_{m}}{E_{f}V_{m}+E_{m}V_{f}}$$

where, *E_f_* and *E_m_* are elastic modulus of fibers and epoxy matrix, respectively; *v_f_* and *v_m_* are Poisson’s ratio for fibers and epoxy matrix, respectively; *V_f_* and *V_m_* are volume fractions for fibers and epoxy matrix, respectively.

In addition, the in-plane shear modulus of the FRP lamina *G*_12_ is given by

(10) $$G_{12}=\frac{G_{f}G_{m}}{G_{f}V_{m}+G_{m}V_{f}}$$

where *G_f_* and *G_m_* are shear modulus of fibers and epoxy matrix, respectively, and these two parameters can be calculated by

(11) $$G_{f}{}_{\left(m\right)}=\frac{E_{f(m)}}{2(1+v_{f(m)})}$$

Under plane stress conditions, the stress-strain relations for the in-plane components of the stress and strain are of the form

(12) $$\left[\begin{array}{c} \varepsilon_{1}\\ \varepsilon_{2}\\ \gamma_{12} \end{array}\right]=\left[\begin{array}{ccc} 1/E_{1} & -v_{12}/E_{1} & 0\\ -v_{12}/E_{1} & 1/E_{2} & 0\\ 0 & 0 & 1/G_{12} \end{array}\right]\{\begin{array}{c} \sigma_{1}\\ \sigma_{2}\\ \tau_{12} \end{array}\}$$

**Figure 5:**
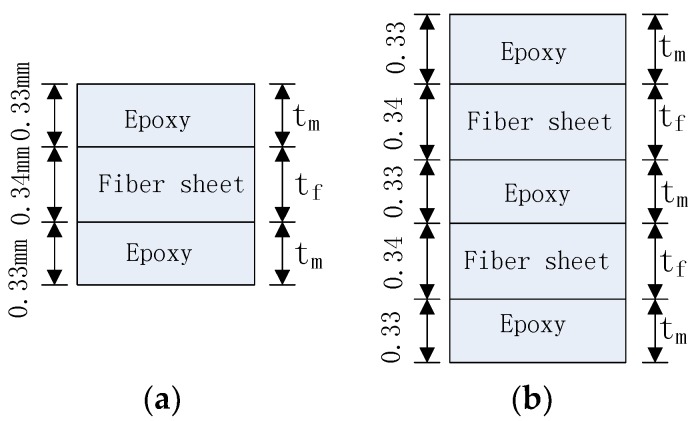
Layer scheme of the FRP lamina in a wet lay-up process: (**a**) One layer of fiber sheet; (**b**) Two layers of fiber sheets.

It should be noted that there are two typical methods forming the FRP lamina: pultruded and wet lay-up in-situ. For the pultruded method, the fibers and the resin (e.g., epoxy) matrix are usually uniformly distributed in the FRP section. As a result, the volumetric ratio of fibers is always a constant regardless of the stiffness of the FRP lamina and can be determined according to the manufacturer’s data. For the wet lay-up process, the FRP lamina can be discretized as dry fiber sheet layers and epoxy layers (Figure 5). Figure 5 shows the thickness profiles in the FRP lamina in cases of one layer and two layers of fiber sheets, respectively. If the nominal thicknesses of each layer of dry fiber sheet and each epoxy layer adjacent to the fiber sheet are assumed to be 0.34 mm and 0.33 mm, respectively (Figure 5), the volumetric ratios of fibers can be obtained as 34% and 40.7%, for one layer and two layers of fiber sheets, respectively.

For element Scheme 1, the following reference values are adopted for the parameters involved in this study: bond length *L* = 150 mm, pure model I fracture energy *G_1f_* = 0.1 N/mm, pure mode II fracture energy *G_2f_* = 0.4 N/mm, peak strength in normal direction *σ^0^* = 2 Mpa, peak strength in tangential direction *τ^0^* = 4 MPa. The FRP lamina is assumed to be formed through the wet lay-up process and the total thickness of one layer of FRP lamina *t* = 1 mm (including the fiber sheet thickness *t_f_* = 0.34 mm and two layers of epoxy resin, with the single layer thickness *t_m_* = 0.33 mm) (see Figure 5a). In addition, the elastic modulus for fibers and epoxy are 250 GPa and 2.50 GPa, respectively, and the Poisson ratio for fibers and epoxy are 0.2 and 0.3, respectively. These values are chosen as realistic values for FRP sheets bonded to a concrete substrate through the wet lay-up procedure (e.g., [8,58]). Thus, the calculated values of *E*_1_, *E*_2_, *v*_12_, *G*_12_ can be used as input parameters in the FE modeling as follows: For the case of one layer of fiber sheet, (Figure 5a), the calculated values are: *E_1_* = 86.65 GPa, *E*_2_ = 3.77 GPa, *v*_12_ = 0.266, *G*_12_ = 1.45 GPa; for the case with two layers of fiber sheets (Figure 5b), which is frequently adopted in a wet lay-up process, *E*_1_ = 103.28 GPa, *E*_2_ = 4.19 GPa, *v*_12_ = 0.259, *G*_12_ = 1.61 GPa.

In Scheme 2 (Figure 4b), the FRP lamina is modeled as 2-node elastic beam with equivalent thickness and equivalent modulus. It should be noted that the upper nodes of cohesive elements should be tied to the bottom of the beam section rather than the central line of the beam element (Figure 4c) to create a similar loading condition at the loading point of FRP (i.e., Point A in Figure 4) for both schemes. Otherwise, an underestimation of the initial stiffness of the peeling force-displacement curves may be caused. The values of the equivalent thickness and equivalent modulus of the beam element were obtained following the principles below:

(13) $$E_{e}t_{e}=E_{m}t_{m}+E_{f}t_{f}$$

(14) $$E_{e}I_{e}=E_{m}I_{m}+E_{f}I_{f}$$

Equation (14) can be rewritten as:

(15) $$E_{e}\frac{t_{e}^{3}}{12}=E_{m}\cdot 2[\frac{t_{m}^{3}}{12}+t_{m}(\frac{t_{f}+t_{m}}{2}{)}^{2}]+E_{f}\frac{t_{f}^{3}}{12}$$

where *E_e_* and *t_e_* are the equivalent elastic modulus and equivalent thickness of the equivalent beam, respectively. For the case of one layer of fiber sheet, the calculated values are *E_e_* = 230.6 GPa, *t_e_* = 0.376 mm; while for the case of two layers of fiber sheets, these two parameters are *E_e_* = 141.1 GPa, *t_e_* = 1.22 mm.

**Figure 6:**
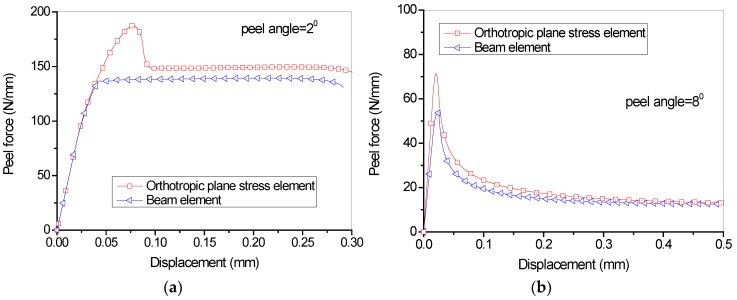
Influence of different element types on the load-displacement curves (1 layer of dry fiber sheets): (**a**) Peel angle = 2 degree; (**b**) Peel angle = 8 degree.

Figure 6 shows the comparison of load-displacement relationship using two different element types for the FRP lamina (consisting of one layer of fiber). Since the focus is placed on the influence of the element types of FRP lamina, the G_1a_/G_1b_ and G_2a_/G_2b_ ratios, which determine the stiffness of the cohesive laws once the fracture energy and peak strength values are fixed, are assumed to equal to 2/3 and 1/9 in all the analyses. Two peel angle 2^0^ and 8^0^, which represent the relatively small angle and large peel angle, respectively, are observed. It can be seen that, generally, the entire debonding process has two phases: the initial phase and the steady peeling phase (Figure 6). The curves present an ascending branch up to a peak peeling force in the initial phase followed by a load drop, and then enter the steady peeling phase which usually has a long plateau. It seems that Scheme 1 and Scheme 2 elements lead to significantly different load-displacement curves. The former schemes usually lead to higher peeling load, particularly the initial peeling load. When peel angle = 8 degree, both schemes lead to relatively close predictions for the load-displacements during the steady peeling phase; when peel angle = 2 degree, element Scheme 2 cannot capture the peak peeling force during the initial phase. This can be attributed to the fact that the elastic isotropic beam elements cannot fully take into account the orthotropic properties of the FRP lamina, though some equivalent measures have been taken in calculating the thickness and elastic modulus of FRP lamina.

**Figure 7:**
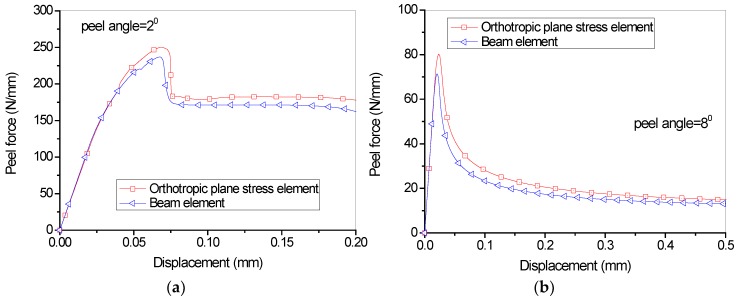
Influence of different element types on the load-displacement curves (2 layers of dry fiber sheets): (**a**) Peel angle = 2 degree; (**b**) Peel angle = 8 degree.

To further understand the influence of different element schemes for FRP lamina on the overall load-displacement curves, FE analyses were also conducted for FRP lamina with 2 layers of fiber sheets (Figure 5b), with other parameters unchanged. The same tendency can be seen from Figure 7, though both element schemes lead to closer predictions. Scheme 2 tends to give relatively lower predictions for the steady peeling force, which can also be attributed to simplification from orthotropic composite lamina to the elastic isotropic beam element, as discussed above. Due to the straightforwardness and simplicity of the calculation of the mechanical parameters for FRP lamina, Scheme 1 is adopted in the following studies.

## 4. Parametric Analyses

### 4.1. Influence of the Unbonded Length of FRP Lamina

**Figure 8:**
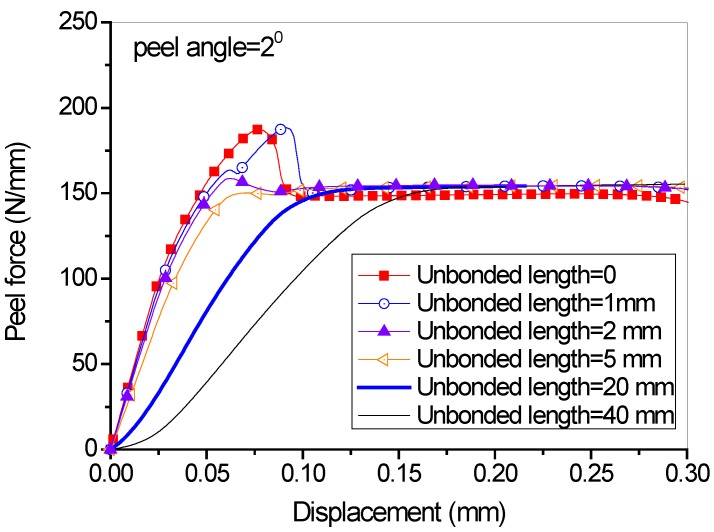
Effect of different unbonded lengths of FRP lamina on the load-displacement behavior (peel angle = 2 degree).

In the studies above, no unbonded length exists between the FRP lamina and the concrete substrate, and the existence of unbonded length was believed to influence the initial peeling and the transition from the initial peeling stage to the steady peeling stage. Different values of unbonded lengths (i.e., 0, 1 mm, 2 mm, 5 mm, 20 mm, 40 mm) were used in the FE analysis. It can be seen from Figure 8 that the unbonded length has a significant effect on the slope of the initial stage as well as the peak peeling force. A longer unbonded length leads to a smooth transition from the initial to the steady peeling stage. However, the values of the steady peeling force for all unbonded lengths are identical to each other. With the increase of the unbonded length, the FRP lamina itself experiences a longer process of elastic deformation before the damage of cohesive elements, affecting the stiffness of the peeling force-displacement curves and the stress-intensity at the crack tip particularly when the unbonded length is short. The unbonded length has a significant effect on the initial peeling behavior of the FRP composites. Once the debonding enters a steady peeling stage, the peeled bond length becomes a part of the unbonded length and the peeling force becomes independent of the unbonded length (i.e., the initial unbonded length + the peeled bond length). In other words, if the initial unbonded length is long enough, the initial peeling force will approach the stable peeling force (i.e., become independent of the initial unbonded length).

### 4.2. Influence of the Peel Angle

**Figure 9:**
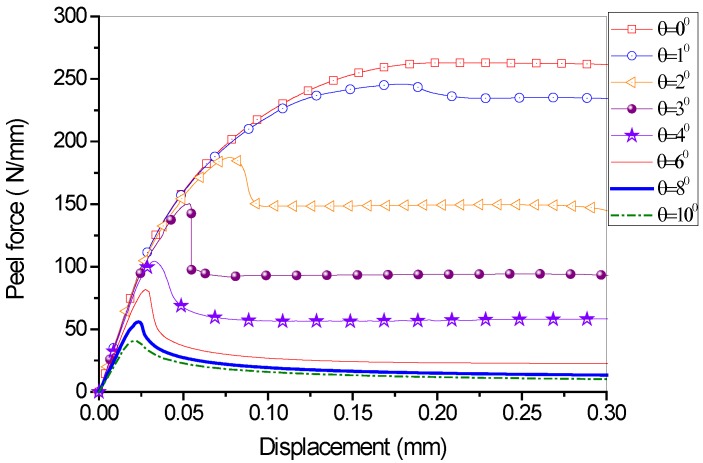
Load-displacement behavior for different peel angle.

**Figure 10:**
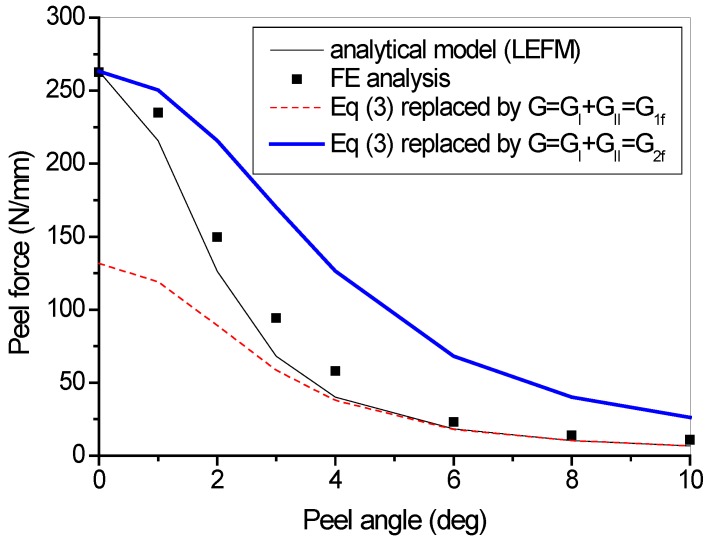
Steady peeling force vs. peel angle.

Parametric studies were conducted on the cases of different peel angles, with all the other parameters the same as those used in the previous studies for one layer of fiber in the FRP lamina. It can be seen from Figure 9 that the steady peeling force decreases with the increase of peel angle. Figure 10 shows the comparison of steady peeling force from the numerical results (i.e., FE modeling) and the analytical model (i.e., LEFM). It can be seen that the numerical modeling and analytical model lead to similar predictions of steady peeling force. However, it has to be pointed out that, in the analytical model, only the failure criterion (i.e., Equation (3) or Equation (4)), which is referred to as the damage evolution law, is taken into account. The DIL (i.e., Equation (5) or Equation (6)), which reflects the shape of cohesive law and will be further discussed in the following sections, cannot be considered. In the analytical model based on the LEFM approach, the cohesive constitutive models (in both normal and tangential directions) are assumed to be linear brittle, with no descending curve after the peak interfacial stress. In contrast, the cohesive constitutive models used in the FE analysis take into account the interfacial softening effects. Thus, the FE analysis is believed to give more reliable results for the steady peeling force.

### 4.3. Influence of the Shape of Cohesive Law

In the analytical model, once the properties (elastic modulus *E* and thickness *t*) of the FRP lamina and the fracture energy of the interface (*G_1f_*, *G_2f_*) are fixed, the peeling force is determined. Under the same fracture energy (*G_1f_*, *G_2f_*) configuration, however, the shape of the cohesive laws may differ significantly. A ratio of *G_a_/G_b_* is introduced here to define the shape of the cohesive laws, where *G_a_* is the initial fracture energy (the area underneath the ascending portion of the cohesive law) and *G_b_* is the area under the descending branch, which is the difference between *G_f_* and *G_a_* (i.e., *G_f_*–*G_a_*).

**Figure 11:**
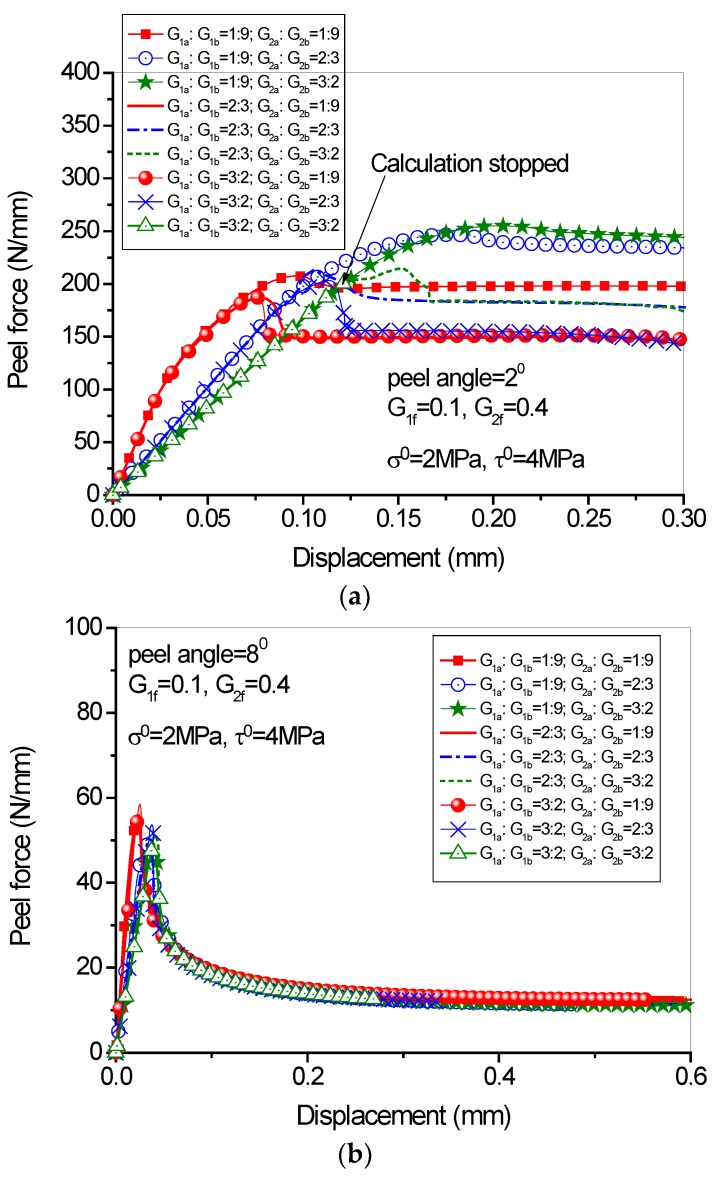
Effect of G_a_/G_b_ ratio on the load-displacement behavior: (**a**) peel angle = 2 degree; (**b**) peel angle = 8 degree.

**Figure 12:**
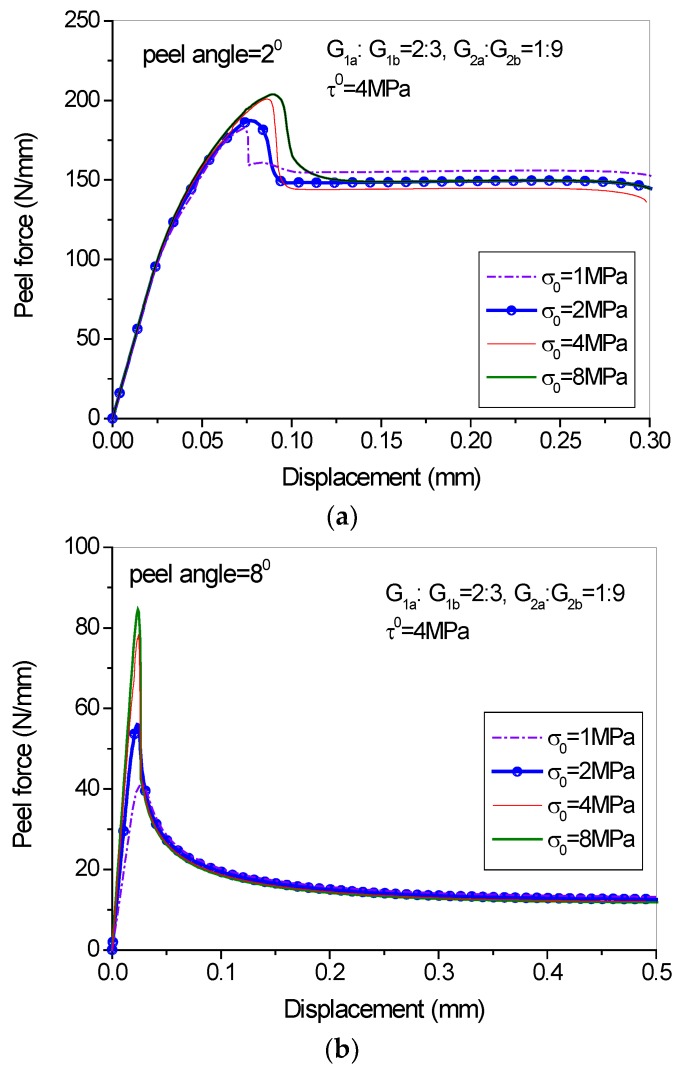
Effect of normal peak strength on the load-displacement behavior: (**a**) peel angle = 2 degree; (**b**) peel angle = 8 degree.

Once the value of *G_1f_* and *G_2f_* is given, the cohesive strength and the value of *G_a_/G_b_* dominate the shape of cohesive laws. It can be seen from Figure 11 that in case of small peel angle range, both mode I and mode II fracture components influence equally the peeling force. In other words, the shape of cohesive laws plays an important role in determining the peeling load-displacement behavior. In the analyses, three values of G_a_/G_b_ (i.e., 1:9, 2:3 and 3:2) for both normal and tangential directions are adopted, leading to 9 sets of combinations for G_1a_/G_1b_ and G_1a_/G_1b_ (Figure 11). Four different normal interfacial strengths (1MPa, 2MPa, 4MPa, 8MPa) are adopted to investigate the influence of the peak cohesive stress on the load-displacement behavior of the peeling test (Figure 12). It is easy to know that the ratio of G_a_/G_b_ in fact reflects the initial stiffness *K* of the interface once the interfacial strength is known.

The analytical results (Figure 11) show that in case of small peeling angle (i.e., 2 degree), changing the initial stiffness of the interface *K* (i.e., G_a_/G_b_) leads to different load-displacement behaviors (both the initial stage and the steady peeling stage; see Figure 11a). While in case of large peel angle (i.e., 8 degree), changing the *K* value does not influence the steady peeling force (Figure 11b). This is because in the latter case the interface failure is mode-I dominated, while in the former case the mode-I and mode-II fracture components have comparable magnitudes. Therefore, in cases of small peeling angle range, selecting cohesive parameters is more essential to the analytical results. In addition, the ascending slope of the load-displacement curves is determined by the initial shear stiffness of the interface (i.e., G_a_/G_b_ value in the tangential direction). However, the time when the interface shear failure occurs is determined by the initial normal stiffness of the interface (i.e., G_a_/G_b_ ratio in the normal direction). One point worth mentioning is that, in the case for which G_1a_/G_1b_ = 3:2, G_2a_/G_2b_ = 3:2 and the peel angle = 2^0^ (see Figure 11a), the calculation stopped before the peak load was obtained. This is because of the setting DIL and DEL in ABAQUS, which will be elaborated in the next section. Usually, the DIL takes into effect first to determine if the peak peel force is attained, and then the DEL plays its role in determining the post-peak behavior. In the above-mentioned case, due to the small initial interface stiffness accompanied with the large values of G_1a_/G_1b_ and G_2a_/G_2b_, the normal stress and shear stress at the interface increase very slowly. As a consequence, the DEL may be met ahead of the DIL. Such a circumstance is allowed in ABAQUS so that the calculation was stopped.

Figure 12 shows that the steady-state peeling load is marginally influenced by the cohesive strength once the ratio of G_a_/G_b_ is determined. However, an increase of the mode-I cohesive strength produces a higher peak load in the initial peel stage and this tendency is more prominent when the peel angle increases (Figure 12b). Parametric studies on the influence of mode II cohesive strength are not conducted here. From Figure 11 and Figure 12 it can be observed that generally the G_a_/G_b_ ratios in the two directions play a significant role for the determination of the load-displacement behavior during both the damage initiation stage and steady peeling stage, while when the G_a_/G_b_ ratios in the two directions are fixed, the cohesive strengths in the normal and tangential directions only influence the peeling force in the damage initiation stage and just have a marginal effect on the steady peeling force.

### 4.4. Influence of the Damage Initiation and Evolution Laws

**Figure 13:**
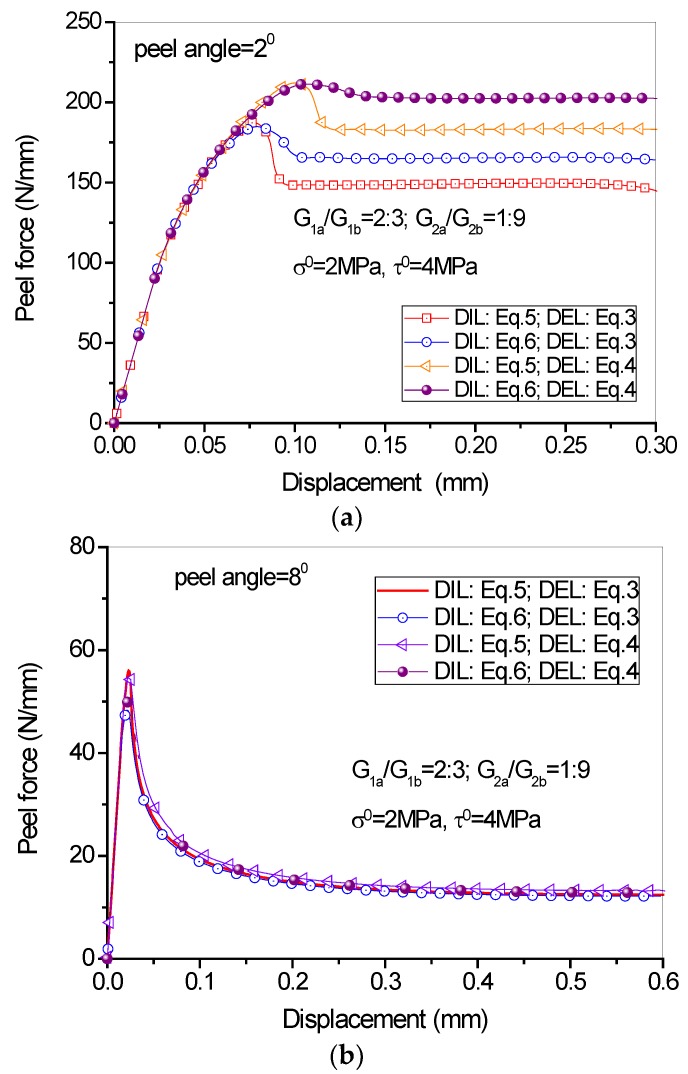
Effect of damage initiation and evolution laws on the load-displacement behavior: (**a**) peel angle = 2 degree; (**b**) peel angle = 8 degree.

The load-displacement curves under different damage initiation and evolution laws are shown in Figure 13. It can be seen from the figure that in case of large peel angle, the DIL and DEL have no significant influence on the peeling process because the debonding is dominated by mode I fracture. When the peel angle is small, however, the DIL and DEL influence significantly the peeling force. The quadric DIL (Equation (6)) leads to a higher peeling force as compared to the maximum cohesive strength DIL (Equation (5)). Besides, the linear DEL (Equation (3)) leads to relatively lower peeling force as compared to the power DEL (Equation (4)).

**Figure 14:**
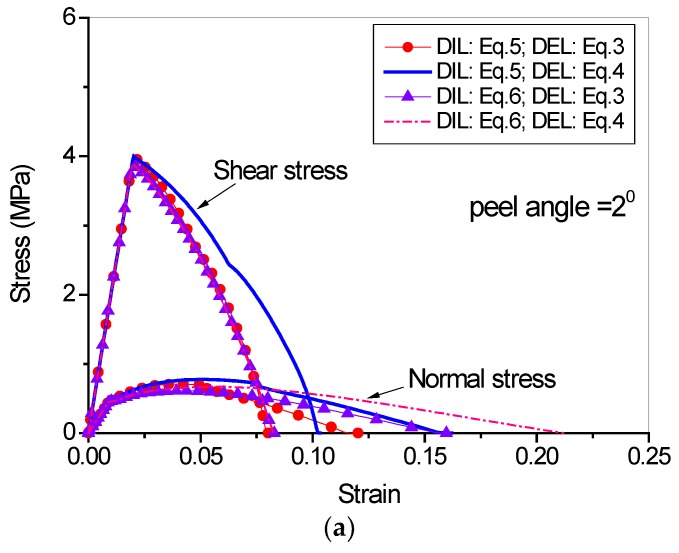

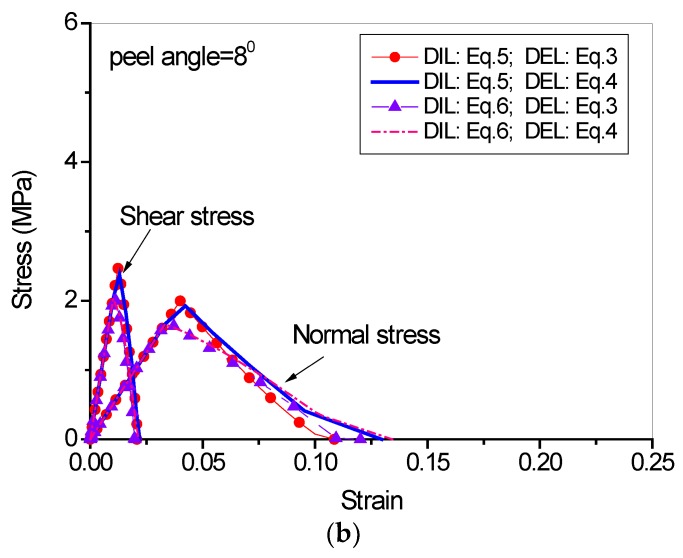
Stress-strain evolutions of the cohesive element under coupled conditions: (**a**) peel angle = 2 degree; (**b**) peel angle = 8 degree.

Figure 14 illustrates the stress-strain evaluations at the interface in cases of small and large peeling angles. It is clearly seen that in case of small peel angle, the interface failure is dominated by shear, while in case of large peel angle, the interface failure is dominated by tension. In addition, it seems that the stress-strain evolutions at the interface are less sensitive to the DIL and DEL in case of large peeling angle (Figure 14b) as compared to the small peeling angle case (Figure 14a).

## 5. Full-Range Debonding Process of an FRP-to-Concrete Interface Subject to Mixed-Mode Loading

**Figure 15:**
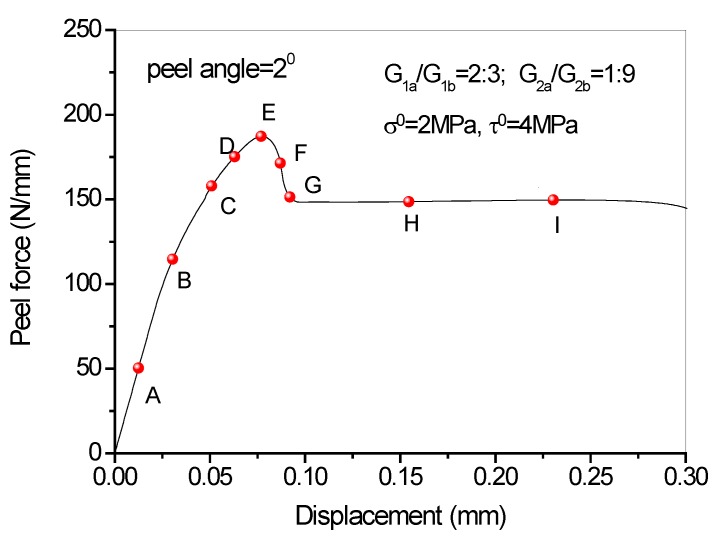
Load-displacement curves when peel angle = 2 degree.

**Figure 16:**
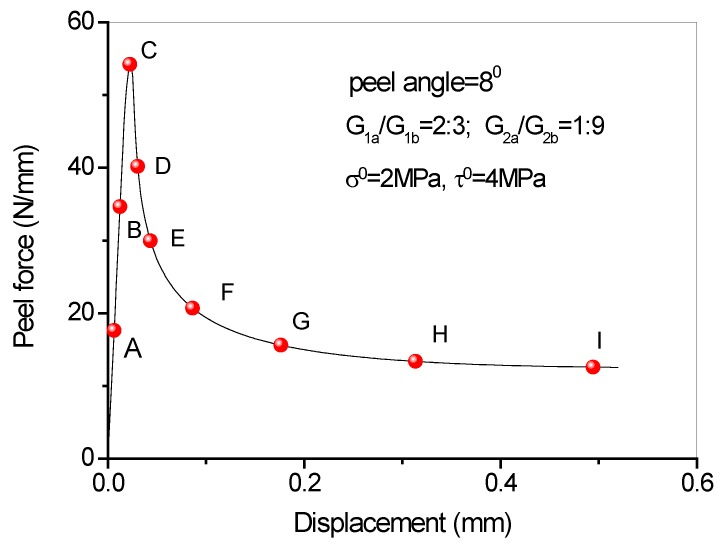
Load-displacement curve when peel angle = 8 degree.

**Figure 17:**
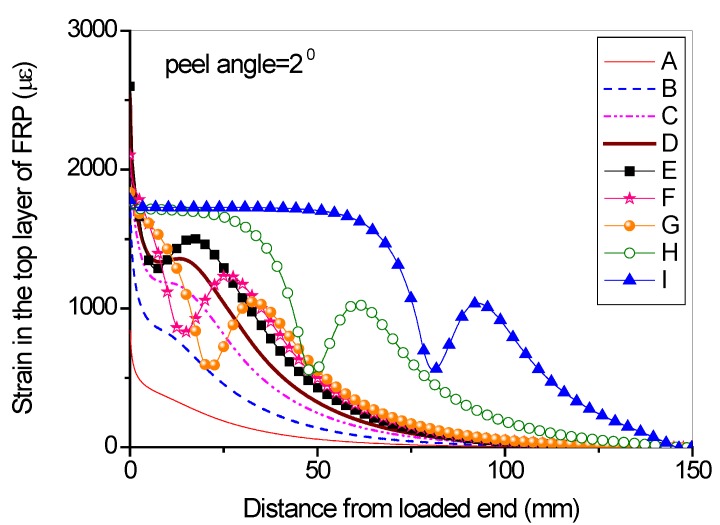
Strain distributions along the surface of FRP lamina when the peel angle = 2 degree.

**Figure 18:**
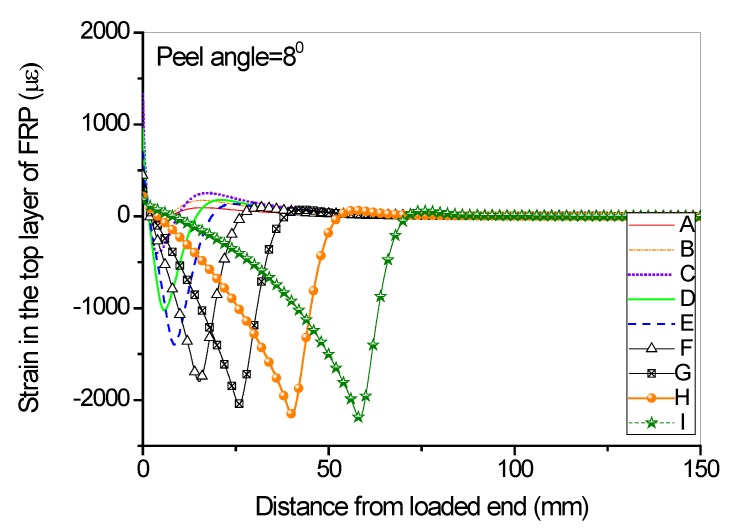
Strain distributions along the surface of FRP lamina when the peel angle = 8 degree.

**Figure 19:**
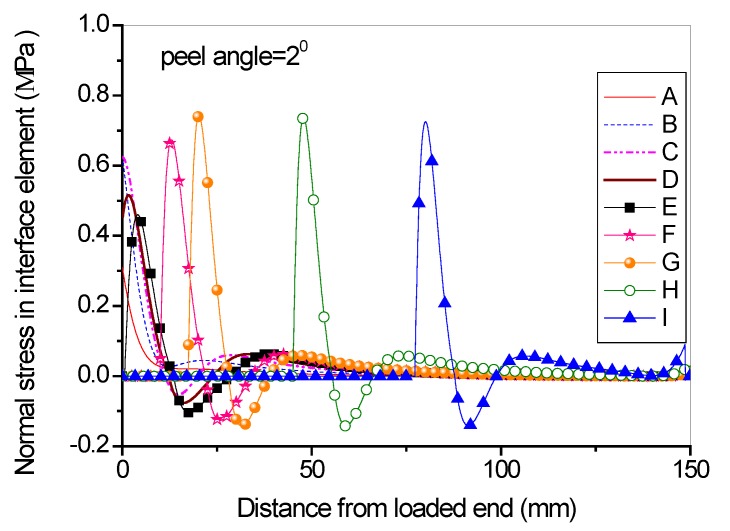
Normal stress distribution along the interface at different load levels when the peel angle = 2 degree.

**Figure 20:**
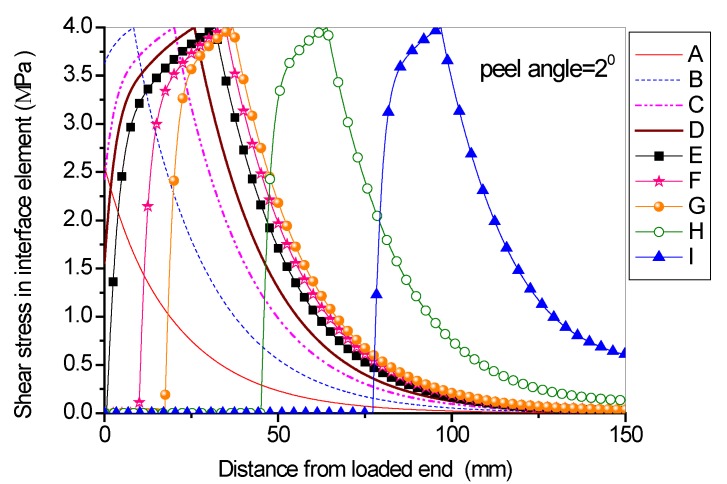
Shear stress distribution along the interface at different load levels when the peel angle = 2 degree.

**Figure 21:**
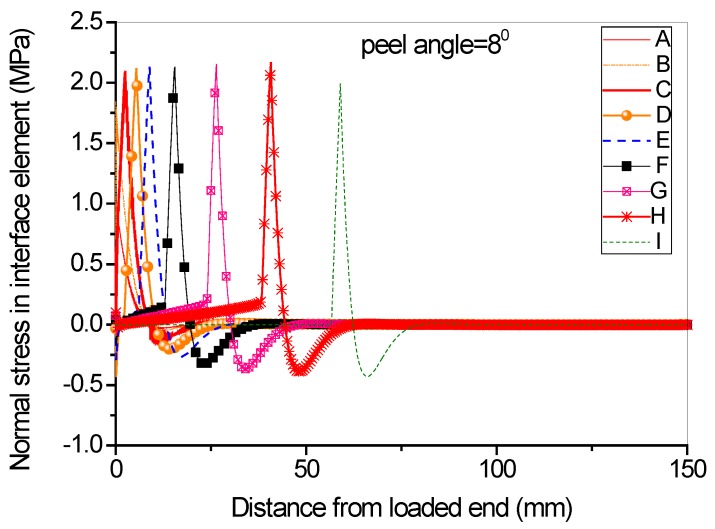
Normal stress distribution along the interface at different load levels when the peel angle = 8 degree.

**Figure 22:**
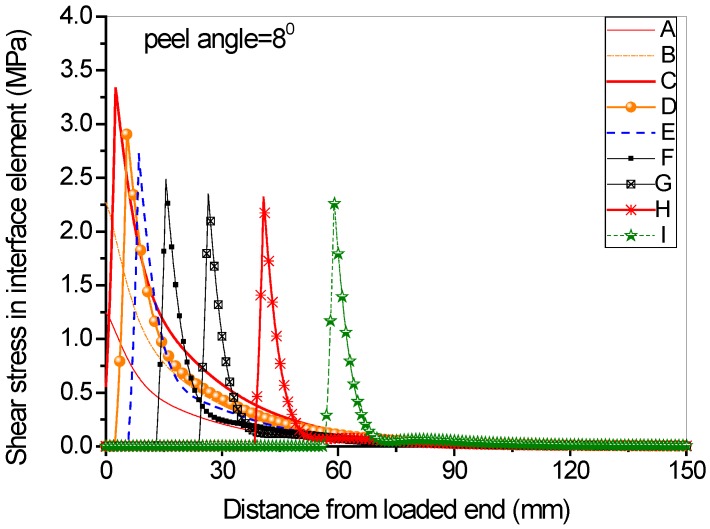
Shear stress distribution along the interface at different load levels when the peel angle = 8 degree.

Full range debonding process of an FRP-to-concrete interface subjected to mixed-model loading has been rarely reported and is thus investigated here. The parameters used in the numerical investigation are as follows: *G_1f_* = 0.1 N/mm, *σ^0^* = 2 MPa, *G_1a_/G_1b_* = 2:3; *G_2f_* = 0.4 N/mm, *τ^0^* = 4 MPa, *G_2a_/G_2b_* = 1:9. Figure 15 and Figure 16 present the peel force-displacement curves for the cases of small peel angle (i.e., 2 degree) and large peel angle (i.e., 8 degree), respectively. The symbols (A, B, C,…, I) in these figures represent different loading levels. Correspondingly, the strain distributions at the surface of FRP lamina are presented in Figure 17 and Figure 18, and normal stress and shear stress distributions in the interface elements are presented in Figure 19, Figure 20, Figure 21 and Figure 22, respectively. The purpose is to facilitate an in-depth understanding of the status of FRP lamina and the interface during the whole peeling process.

From the strain distributions at the surface of FRP lamina, it can be seen that, during the peeling process, the FRP lamina is subjected to a local bending status, which is indicated by the variation of the strains along the lamina (Figure 17 and Figure 18). When the peel angle is large, the surface strains of FRP lamina even exhibit minus values, indicating significant local bending in the lamina (Figure 18). When the peeling angle is small (i.e., 2 degree), the normal stress is relatively small, with the maximum strain value of 0.8 MPa in tension. The normal stress exhibits compression at some locations due to the local bending effects (Figure 19). On the other hand, the shear debonding propagates along the interface which is similar to that observed in the mode II tests of FRP-to-concrete interfaces (Figure 20). In contrast, when the peel angle is large (8 degree), the debonding process is driven by the mode I fracture and the peak normal stress shifts along the debonding direction of the FRP lamina (Figure 21). These results are similar with the observed strain variation tendency as reported by Dai et al. [8].

## 6. Conclusions

Comprehensive FE modeling has been conducted to reveal the debonding mechanisms of FRP-to-concrete interface subjected mix-mode loading, which is realized through simulating a typical peel test. Through the implementation of the cohesive zone models coupled in normal and tangential directions and appropriate simulation of the FRP lamina, the following conclusions can be drawn:

1. Regardless of the peel angle, the entire debonding process of FRP-to-concrete interfaces consists of two phases: the initiation phase and the steady-state peeling phase. With the increase of peel angle (e.g., >4 degree), the debonding process shifts rapidly from mode II-dominated to mode I-dominated. Below this angle, the mode I and mode II components have comparable amplitudes, so their coupling effect is very significant.
2. The orthotropic plane stress element scheme seems to be most appropriate for the simulation of the FRP lamina during the mix-mode debonding of FRP-to-concrete interfaces and the mechanical properties can be determined based on the composite mechanics.
3. In case of large peel angle (e.g., >4 degree), both the DIL and the DEL have an insignificant influence on the peeling process; while, when the peel angle is relatively small, both the DIL and DEL have a significant influence on the peeling force. The quadric DIL leads to higher peeling force than the maximum cohesive strength DIL. Besides, the power DEL leads to higher peeling force than the linear DEL.
4. Analyses of the full-range mixed-mode debonding process helps to achieve a thorough understanding of phenomena observed in the peeling test of an FRP-to-concrete interface, in terms of the global load-displacement curves, and the local strain distributions along the FRP lamina length as well as the stress-strain evolution history at different load levels.

## References

[1] Teng J.G., Chen J.F., Smith S.T., Lam L. (2002). FRP-Strengthened RC Structures.

[2] Hollaway L.C., Teng J.G. (2008). Strengthening and Rehabilitation of Civil Infrastructures Using Fibre-Reinforced Polymer (FRP) Composites.

[3] Seyhan E.C., Goksu C., Uzunhasanoglu A., Ilki A. (2015). Behavior of substandard RC columns retrofitted with embedded aramid fiber reinforced polymer (AFRP) reinforcement. Polymers.

[4] Lee S.K., Lee Y.B., Seo Y.S. (2016). A seismic strengthening technique for reinforced concrete columns using sprayed FRP. Polymers.

[5] Ueda T., Dai J.G. Interface of fiber reinforced polymer laminates externally bonded to concrete substrate: From test methods to bond modeling. Proceedings of the International Symposium on Bond Behaviour of FRP in Structures (BBFS 2005).

[6] Wu Z.S., Yuan H., Asakura T., Yoshizawa H., Kobayashi A., Kojima Y. (2005). Peeling behavior and spalling resistance of bonded bidirectional fiber reinforced polymer sheets. J. Compos. Constr..

[7] Wu Z.S., Yuan H., Kojima Y., Ahmed E. (2005). Experimental and analytical studies on peeling and spalling resistance of unidirectional FRP sheets bonded to concrete. Compos. Sci. Technol..

[8] Dai J.G., Ueda T., Sato Y. (2007). Bonding characteristics of fiber-reinforced polymer sheet-concrete interfaces under dowel load. J. Compos. Constr..

[9] Dai J.G., Cao Y.B. Debonding Behavior of Skew FRP-Bonded Concrete Joints. Proceedings of the 5th International Conference on FRP Composites in Civil Engineering (CICE2010).

[10] Taljsten B. (1997). Defining anchor lengths of steel and CFRP plates bonded to concrete. Int. J. Adhes. Adhes..

[11] Chen J.F., Teng J.G. (2001). Anchorage strength models for FRP and steel plates bonded to concrete. J. Struct. Eng..

[12] De Lorenzis L., Miller B., Nanni A. (2001). Bond of fiber-reinforced polymer laminates to concrete. Aci. Mater. J..

[13] Yuan H., Wu Z., Yoshizawa H. (2001). Theoretical solutions on interfacial stress transfer of externally bonded steel/composite plates. J. Struct. Mech. Earthq. Eng..

[14] Dai J.G., Ueda T., Sato Y. (2005). Development of the nonlinear bond stress-slip model of fiber reinforced plastics sheet-concrete interfaces with a simple method. J. Compos. Constr..

[15] Lu X.Z., Teng J.G., Ye L.P., Jiang J.J. (2005). Bond-slip models for FRP sheets/plates bonded to concrete. Eng. Struct..

[16] Yao J., Teng J.G., Chen J.F. (2005). Experimental study on FRP-to-concrete bonded joints. Compos. Part B.

[17] Gao W.Y., Teng J.G., Dai J.G. (2012). Effect of Temperature Variation on the Full-Range Behavior of FRP-to-concrete Bonded Joints. J. Compos. Constr..

[18] Au C., Büyüköztürk O. (2006). Debonding of FRP plated concrete: A tri-layer fracture treatment. Eng. Fract. Mech..

[19] Biscaia H.C., Chastre C., Silva M.A.G. (2013). Linear and nonlinear analysis of bond-slip models for interfaces between FRP composites and concrete. Compos. Part B.

[20] Biscaia H.C., Chastre C., Silva M.A.G. (2012). Double shear tests to evaluate the bond strength between GFRP/concrete elements. Compos. Struct..

[21] Zhou Y.W., Wu Y.F., Yun Y. (2010). Analytical modeling of the bond-slip relationship at FRP-concrete interfaces for adhesively-bonded joints. Compos. Part B.

[22] Zhou Y.W., Fan Z.H., Du J., Sui L.L., Xing F. (2015). Bond Behavior of FRP-to-Concrete Interface under Sulfate Attack: An Experimental Study and Modeling of Bond Degradation. Constr. Build. Mater..

[23] Qiao P.Z., Xu Y.W. (2004). Evaluation of fracture energy of composite-concrete bonded interfaces using three-point bend tests. J. Compos. Constr..

[24] Coronado C.A., Lopez M.M. (2008). Experimental characterization of concrete-epoxy interfaces. J. Mater. Civil. Eng..

[25] Zheng J.J., Dai J.G., Fan X.L. (2016). Fracture analysis of FRP-plated notched concrete beam subjected to three-point bending. J. Eng. Mech..

[26] Dai J.G., Ueda T., Hasan M., Sato Y. (2003). Mode I fracture behaviors of FRP-concrete interfaces. Proc. Jpn. Concr. Inst..

[27] Karbhari V.M., Engineer M. (1996). Investigation of bond between concrete and composites: Use of a peel test. J. Reinf. Plast. Comp..

[28] Wan B.L., Sutton M.A., Petrou M.F., Harries K., Ning U. (2004). Investigation of bond between fiber reinforced polymer and concrete undergoing global mixed mode I/II loading. J. Eng. Mech..

[29] Pan J., Leung C.K.Y. (2007). Debonding along the FRP-concrete interface under combined pulling/peeling effects. Eng. Fract. Mech..

[30] Alam M.S., Kanakubo T., Yasojima A. (2012). shear-peeling bond strength between continuous fiber sheet and concrete. Aci. Struct. J..

[31] Mohammadi T., Wan B., Harries K.A., Sweriduk M.E. (2017). Bond behavior of FRP–concrete in presence of intermediate crack debonding failure. J. Compos. Constr..

[32] Ghorbani M., Mostofinejad D., Hosseini A. (2017). Bond behavior of CFRP sheets attached to concrete through EBR and EBROG joints subject to Mixed-Mode I/Ii loading. J. Compos. Constr..

[33] Wang J. (2007). Cohesive zone model of FRP-concrete interface debonding under mixed-mode loading. Int. J. Solids. Struct..

[34] Wang J. (2007). Cohesive-bridging zone model of FRP-concrete interface debonding. Eng. Fract. Mech..

[35] Wang J., Zhang C. (2008). Nonlinear fracture mechanics of flexural-shear crack induced debonding of FRP strengthened concrete beams. Int. J. Solids. Struct..

[36] Dai J.G., Wan B., Yokota H., Ueda T. (2009). Fracture criterion for carbon fiber reinforced polymer sheet to concrete interface subjected to coupled pull-out and push-off actions. Adv. Struct. Eng..

[37] De Lorenzis L., Zavarise G. (2008). Modeling of mixed-mode debonding in the peel test applied to superficial reinforcements. Int. J. Solids. Struct..

[38] De Lorenzis L., Zavarise G. (2009). Cohesive zone modeling of interfacial stresses in plated beams. Int. J. Solids. Struct..

[39] De Lorenzis L., Zavarise G. (2009). Interfacial stress analysis and prediction of debonding for a thin plate bonded to a curved substrate. Int. J. Nonlin. Mech..

[40] De Lorenzis L., Zavarise G. (2010). Debonding analysis of thin plates from curved substrates. Eng. Fract. Mech..

[41] Dimitri R., Trullo M., De Lorenzis L., Zavarise G. (2015). Coupled cohesive zone models for mixed-mode fracture: A comparative study. Eng. Fract. Mech..

[42] Michels J., Zile E., Czaderski C., Motavalli M. (2014). Debonding failure mechanisms in prestressed CFRP/epoxy/concrete connections. Eng. Fract. Mech..

[43] Kishi N., Zhang G.F., Mikami H. (2005). Numerical cracking and debonding analysis of RC beams reinforced with FRP sheet. J. Compos. Constr..

[44] Niu H., Karbhari V.M., Wu Z. (2006). Diagonal macro-crack induced debonding mechanisms in FRP rehabilitated concrete. Compos. Part B.

[45] Lee J.H., Chacko R.M., Lopez M.M. (2010). Use of mixed-mode fracture interfaces for the modeling of large-scale FRP-strengthened beams. J. Compos. Constr..

[46] Yin J., Wu Z.S., Andou T., Kato K. Modeling of mixed-mode debonding along FRP-concrete bond interface. Proceedings of the World Conference on Computational Mechanics.

[47] Alfano G., Crisfield M.A. (2001). Finite element interface models for the delamination analysis of laminated composites. Mechanical and computational issues. Int. J. Numer. Meth. Eng..

[48] Camanho P.P., Davila C.G. Mixed-Mode Decohesion Elements for the Simulation of Delamination in Composite Materials.

[49] Kendall K. (1971). Adhesion and surface energy of elastic solids. J. Phys. D Appl. Phys..

[50] Kendall K. (1971). Thin-film peeling-elastic term. J. Phys. D Appl. Phys..

[51] Kim K., Aravas N. (1988). Elastoplastic analysis of the peel test. Int. J. Solids. Struct..

[52] Hutchinson J.W., Suo Z. (1992). Mixed-mode cracking in layered materials. Adv. Appl. Mech..

[53] Kafkalidis M.S., Thouless M.D., Yang Q.D., Ward S.M. (2000). Deformation and fracture of adhesive layers constrained by plastically-deforming adherends. J. Adhes. Sci. Technol..

[54] Thouless M.D., Jensen H.M. (1992). Elastic fracture-mechanics of the peel-test geometry. J. Adhesion.

[55] Suo Z.G., Hutchinson J.W. (1990). Interface crack between two elastic layers. Int. J. Fracture.

[56] ABAQUS (2008). ABAQUS Standard User’s Manual, Volumes I-III, Version 6.8.

[57] Yu T., Teng J.G., Chen J.F. (2009). Mechanical properties of FRP composites. ICE Manual of Construction Materials.

[58] (2004). CNR-DT 200/2004: Guide for the Design and Construction of Externally Bonded FRP Systems for Strengthening Existing Structures-Materials, RC and PC Structures, Masonry Structures.

